# 可切除Ⅲa/N2期非小细胞肺癌治疗模式探讨

**DOI:** 10.3779/j.issn.1009-3419.2019.02.06

**Published:** 2019-02-20

**Authors:** 子宜 许, 镨元 邢, 迪 马, 以香 朱, 建明 应, 峻岭 李

**Affiliations:** 1 100021 北京，国家癌症中心/国家肿瘤临床医学研究中心/中国医学科学院北京协和医学院肿瘤医院 National Cancer Center/National Clinical Research Center for Cancer/Caner Hospital, Chinese Academy of Medical Sciences and Peking Union Medical College, Beijing 100021, China; 2 550004 贵阳，贵州医科大学附属肿瘤医院（贵州省肿瘤医院） Affiliated Hospital of Guizhou Medical University, Guizhou Province Tumor Hospital, Guiyang 550004, China

**Keywords:** 可切除Ⅲa/N2期非小细胞肺癌, 化疗, 放疗, 手术, Resectable Ⅲa/N2 non-small cell lung cancer, Chemotherapy, Radiotherapy, Surgery

## Abstract

临床可切除Ⅲa/N2期非小细胞肺癌（non-small cell lung cancer, NSCLC）的治疗模式一直存在争议，化疗、放疗、手术等治疗手段都有可改善其生存的报道，靶向治疗和免疫治疗的研究也有较多新进展，联合治疗的选择更是很多临床试验研究的重点。联合治疗包括化疗联合手术或放疗，以及化疗、放疗和手术三种治疗的联合。对于NSCLC最佳治疗模式目前尚无定论。本文通过对多项Ⅱ期、Ⅲ期临床试验及*meta*分析、个案报道的综述回顾，比较不同治疗方式对临床可切除Ⅲa/N2期NSCLC的生存影响，结果显示多学科治疗中手术对于延长患者生存是必要的选择，而新辅助化疗后联合手术并不优于联合放疗。依据肿瘤大小、淋巴结累及情况等进行个体化治疗是未来值得进一步探索的方向。

## Abstract (en)

Standard treatment for resectable Ⅲa/N2 non-small-cell lung cancer (NSCLC) is still under debate. Optional treatments include chemotherapy, radiotherapy and surgery, other options include target therapy and immunotherapy. Multidisciplinary treatment has therefore been emphasized by various clinical trials, including bimodality strategy which has been defined as chemotherapy plus surgery or chemotherapy plus radiotherapy, and trimodality treatment which refers to chemotherapy plus surgery and radiotherapy. However, there is still no consensus on the optimal strategy on treating resectable Ⅲa/N2 NSCLC. Therefore, we reviewed a series of phase Ⅱ and Ⅲ clinical trials as well as some *meta*-analyses and case reports to compare the efficacy of different strategies on survival of cN2 NSCLC, and concluded that for resectable Ⅲa/N2 NSCLC surgery is recommended, and that strategy of chemotherapy plus surgery may not achieve better survival than that of chemotherapy plus radiotherapy. Size of tumor as well as lymph nodes should be taken into account when choosing optimal therapy, so that promising individualized strategy could be given to patients with resectable stage Ⅲa/N2 NSCLC.

肺癌是我国乃至全世界死亡率最高的恶性肿瘤，非小细胞肺癌（non-small cell lung cancer, NSCLC）占肺癌的比例接近85%^[1]^，其中约1/3的NSCLC患者在首次确诊时为局部晚期肿瘤（Ⅲa期/Ⅲb期），虽然潜在可治愈机会，但仍有60%以上的患者出现复发/转移以致死亡^[2]^。不同于早期Ⅰ期/Ⅱ期NSCLC患者有较高的手术治愈率，Ⅲ期患者在术后仍有复发和死亡风险^[3]^。临床可切除Ⅲa/N2期NSCLC的治疗应该是多学科治疗，两种治疗方式的联合包括化疗联合手术或放疗，三种治疗方式的联合指化疗联合放疗和手术，最合理的选择还在探索中。化疗联合手术的疗效是否优于化疗联合放疗，三种治疗方式联合的疗效是否优于化疗联合手术或放疗，最终的选择应包含方案对患者生存、生活质量的影响，以及手术方式、化疗方案等可行性和安全性的考虑。

## 1 手术方式

手术切除在部分Ⅲa/N2期患者的多学科综合治疗方案中都是一个重要治疗方法。手术是否能完全切除病灶，对于病人整体预后而言都是重要的参数，因此，同侧纵隔淋巴结转移NSCLC预后和治疗因淋巴结侵袭性不同而有很大差异，目前根据是否可切除将Ⅲa/N2期NSCLC分为三类^[4]^，一类是明确可根治性切除，一类是有潜在切除的可能性，这类患者有不完全切除的风险，还有一类患者明确不可切除。对于明确可切除N2期NSCLC患者，标准治疗为手术切除，术后辅助化疗或化疗加放疗；不可切除N2期的标准治疗为同步放化疗，同时，免疫治疗用于不可切除N2期NSCLC中同步放化疗后改善生存的作用也得到了更多关注。不完全切除的姑息性手术对于改善生存无益，并且手术完成质量、淋巴结的清扫均会影响预后^[5]^。随着TNM分期的更新修改，N2期NSCLC人群中的预后差别较大，微小的N2淋巴结与淋巴结旁浸润的患者有截然不同的预后^[6]^。Betticher等^[7]^进行的一项Ⅱ期临床研究对病理证实N2期NSCLC患者进行多西他赛-顺铂诱导化疗后手术切除，发现化疗后分期持续为ypN2的患者与化疗后分期降级的患者进行手术切除预后明显不同，从而推荐手术只适用于后者。相反，有研究^[4, 8]^认为化疗后持续为N2期的患者较化疗后降级为N0或N1的患者而言5年生存率并无明显差异，二者均可在化疗后的完全切除手术中获益。Massard等^[6]^发表的综述认为在Ⅲa/N2期NSCLC患者的多学科综合治疗中，根治性切除加淋巴结清扫术的治疗是合理的，即使对于持续N2期患者，新辅助化疗后的根治性手术切除同样是必要的，并且尽早手术获益更大。

## 2 综合治疗

手术联合放化疗的治疗方案是否优于化疗联合放疗，或化疗联合手术的治疗模式，是多项Ⅲ期临床试验所探讨的重点。2008年ANITA试验在术后化疗对比安慰剂的基础上加入术后化疗（PORT）对生存影响的研究，发现在联合长春瑞滨加顺铂化疗组，pN2期患者术后PORT联合化疗比单独术后化疗获得更长的中位生存（47.4个月*vs* 23.8个月）和更高的5年存活率（47.4% *vs* 34%），表明术后放疗联合化疗pN2期NSCLC的获益较其他病理分期更明显^[9]^。

2015年在*Lancet*杂志上发表的一项前瞻性研究对比了诱导化疗后进行加速放疗及手术组与诱导化疗后手术组的效果^[10]^，研究采用了无事件生存期（event-free survival, EFS）为主要生存终点。有研究者认为，在NSCLC患者化疗的研究中，EFS可作为总生存期（overall survival, OS）的替代终点，而在局部晚期肺癌的化疗与放疗研究中可作为无进展生存期（progression-free survival, PFS）的替代终点^[11]^。该前瞻性研究在23个研究中心共纳入232例患者并进行随机分组，117例患者在化疗序贯放疗组中接受了3个周期顺铂加多西他赛联合放疗（44 Gy），115例患者在单独化疗组。随后所有患者均接受手术。两组患者的EFS相似，OS也相似，化放疗组EFS为12.8个月（95%CI: 9.7-22.9），OS为37.1个月（95%CI: 22.6-50.0），而化疗组EFS为11.6个月（95%CI: 8.4-15.2），OS为26.2个月（95%CI: 19.9-52.1），因此该研究认为化疗联合根治性手术可达到与三种治疗方法相似的效果。但由于该研究样本量较小，不足以对这两种治疗方案进行非劣性分析，且其采用的化疗序贯加速放疗的方式并非目前常用的同步放化疗，因此不能直接确定这类分期患者的最佳治疗方案。一项回顾性研究在对比了249例Ⅲa期NSCLC患者放化疗后是否进行手术的生存情况，放化疗后接受肺叶切除的中位生存优于单独放化疗（分别为39个月和22个月，*P*=0.038），但接受全肺切除者中位生存与单独放化疗相比无明显差异（分别为28个月和22个月，*P*=0.534）^[12]^。这个结果与Intergroup 0139 Ⅲ期临床试验研究结果一致，全肺切除并不作为常规推荐^[13]^。在INT0139试验中，共纳入396例T1-3pN2M0患者，接受同步PE方案（顺铂联合依托泊苷）化疗和放疗（45 Gy），在无进展的情况下，一组进行手术切除后再接受2周期PE方案化疗，另一组继续放疗至61 Gy再接受2周期PE方案化疗。综合治疗的主要研究终点中位OS分别为23.6个月和22.2个月（*P*=0.24, HR=0.87, 95%CI: 0.7-1.1），无明显差异。三种治疗方式的联合较两种治疗方式显著提高了次要终点PFS和5年OS，分别为12.8个月和10.5个月（*P*=0.017, HR=0.77, 95%CI: 0.62-0.96），以及22.4%和11%。虽然该研究并未比较全肺切除和肺叶切除OS，但全肺切除的并发症导致手术组的治疗相关死亡率升高。另外，在亚组分析中，90例放化疗后行肺叶切除的患者OS较单纯放化疗患者OS延长，分别为33.6个月和21.7个月（*P*=0.002）。更多的Ⅲ期研究亟待开展，三种治疗联合的有效性和安全性仍待证明。

化疗联合手术或放疗的模式究竟何者更有利于改善生存，有研究认为二者并无明显差异。单纯手术切除的5年存活率为7%-24%，术前化疗可将存活率提高至17%-36%，而以铂类为基础的化疗联合放疗的5年存活率为15%。2006年多中心大型ANITA Ⅲ期临床研究在Ⅰb期-Ⅲa期NSCLC患者完全切除手术后对比使用长春瑞滨联合顺铂和安慰剂，发现化疗明显改善患者生存（中位生存期65.7个月*vs* 43.7个月，*P*=0.017），而亚组分析发现在Ⅱ期和Ⅲa期效果更明显，为Ⅲa期NSCLC完全切除后使用铂类为基础的化疗提供了新的证据^[14]^。后续ANITA临床试验在对照组加入PORT治疗，发现pN2期NSCLC患者中结果与上述术后化疗组加入PORT相似，都明显取得较长生存获益（5年存活率21.3% *vs* 16.6%），提示pN2期患者术后放疗可带来获益^[9]^。

欧洲癌症治疗研究组织EORTC 08941多中心前瞻性Ⅲ期随机临床试验对比了cN2期NSCLC患者诱导化疗后手术与放疗的效果，纳入579例患者，在3个周期含铂方案诱导化疗后达到61%的缓解率（95%CI: 0.57-0.65），其中332例患者随机分配到手术组（167例）和放疗组（165例），在手术组中154例（92%）接受手术的患者术后30 d死亡率为4%，其中全肺切除的死亡率为7%。手术组和放疗组的中位OS分别为16.4个月和17.5个月，5年生存率分别为15.7%和14%（*P*=0.596, HR=1.06, 95%CI: 0.84-1.35），表明二者生存无明显差异，从而认为诱导化疗后不必要进行手术，可选择副作用更小的化疗方法^[15]^。一篇纳入了包括EORTC 08941在内多项Ⅲ期临床试验的*meta*分析比较化疗加手术的效果与化疗加放疗的效果，发现两种治疗方式无差异（HR=1.01, *P*=0.954）^[16]^。但在该EORTC研究中手术组有50%的患者未进行完全性R0清除，即完整切除原发灶，镜下切缘阴性，系统淋巴结清扫且要求最高淋巴结，使实验结论遭到质疑。2015年发表的Ⅲ期ESPATUE临床试验纳入246例可手术Ⅲa/N2期和特定Ⅲb期患者，经诱导化疗和同步放化疗后比较根治性手术和根治性放化疗的疗效。诱导化疗为3周期紫杉醇加顺铂，同步放化疗为长春瑞滨加顺铂联合加速超分割放疗（45 Gy）。在246例入组患者中有161例（65.4%）在诱导治疗后评估为可切除，随机分为手术组和放化疗组。手术组有81%行R0完全切除术，放化疗组放疗剂量增至65 Gy-71 Gy。结果表明，两组5年OS分别为44%和40%，PFS分别为35%和32%，无统计学差异，因此可认为两种治疗模式均取得令人满意的疗效^[17]^。

## 3 辅助靶向治疗

虽然靶向治疗并未被纳入Ⅲa/N2期NSCLC标准治疗方案，但国内外已有多中心Ⅲ期临床研究报道手术后使用表皮生长因子受体酪氨酸激酶抑制剂（epidermal growth factor receptor-tyrosine kinase inhibitor, EGFR-TKI）的安全性和有效性，靶向治疗或可获得与化疗相似的疗效，甚至较化疗后生存更有获益。2014年Ⅱ期临床研究SELECT试验纳入100例Ⅰa期-Ⅲa期经手术切除和术后标准化疗和/或放疗治疗的*EGFR*基因突变NSCLC患者，继续口服厄洛替尼治疗，发现其2年无病生存期（disease-free survival, DFS） > 85%明显长于历史对照（76%），在停药后12个月复发率最高，提示延长靶向治疗的疗程可能更有效^[18]^。该研究尚未有中位DFS和OS的数据。2015年发表的Ⅲ期临床研究RADIANT试验纳入973例Ⅰb期-Ⅲa期完全切除的*EGFR*基因突变NSCLC患者，随机分组对照口服厄洛替尼和安慰剂的疗效，结果表明在161例m*EGFR*表达阳性的亚组患者中，厄洛替尼组达到更长的中位DFS（46.4个月*vs* 28.5个月，*P*=0.034），但由于分层检验该DFS延长并无统计学意义，各分期亚组中也并未发现明显延长的PFS^[19]^。与前两个大型临床试验的对照以及纳入病人的肿瘤分期不同，2018年在中国进行的一项大型多中心Ⅲ期临床试验ADJUVANT研究纳入222例Ⅱ期-Ⅲa（N1-N2）期完全切除的*EGFR*基因突变NSCLC患者，随机分组进行口服吉非替尼治疗或长春瑞滨联合顺铂化疗，结果发现术后靶向治疗的中位DFS比术后化疗明显延长（28.7个月*vs* 18个月，*P*=0.005, 4），3年DFS也较之延长但无显著差异（34% *vs* 27%, *P*=0.37），且靶向治疗的不良事件更少，3级或以上不良事件的发生明显低于化疗（12.3% *vs* 48.3%），可明显改善患者生存质量^[20]^。亚组分析显示所有类型均可从中获益，但长期生存情况尚待进一步观察。与ADJUVANT研究结果具有同样提示作用的还有一个针对中国患者的多中心随机Ⅱ期EVAN临床研究，比较厄洛替尼与含铂化疗对于R0切除后Ⅲa期*EGFR*突变NSCLC患者的疗效与安全性，结果提示术后靶向治疗组的2年DFS和3年DFS比化疗组明显延长（81.4% *vs* 44.6%, 54% *vs* 19.8%, *P* < 0.05），直接证实靶向治疗对于有基因突变的Ⅲ期NSCLC患者可行且有益^[21]^。同样，其OS数据尚在研究统计中，但术后靶向治疗方案的生存获益可见明显趋势。

靶向治疗作为术前治疗对手术疗效的影响也有相关个案报道。Dumont等^[22]^曾报道1例术前证实为*ALK*重排的NSCLC患者在接受2周期化疗后评估其治疗获益和风险，改为4周克唑替尼治疗（250 mg，每日2次）后影像学评估缓解，继续治疗3周后进行肺叶切除和淋巴结清扫术，病理分期为yp T1a N2期。术后行卡培他滨和培美曲塞化疗联合66 Gy放疗，达到18个月无病生存。但在该个例中，靶向治疗并未在病理学证实其治疗效果，只是术前2周期化疗未取得缓解时的另一种方案选择。Imanishi等^[23]^近期报道了1例术前影像学证实为cN2期的*ALK*重排NSCLC患者接受了3个月的艾乐替尼后，复查影像学证实缓解，进行完全切除手术后病理证实治疗有效（切除组织中可见 < 10%活肿瘤细胞）。术后5个月，患者未进行任何治疗，并达到了无复发生存。尽管有相关报道提示术前靶向治疗可能有降低局部晚期NSCLC分期的作用，靶向治疗对于提高手术安全性的作用还需更多的大型研究证实。

## 4 其他治疗

虽然一些Ⅲ期临床试验证实，以顺铂或卡铂为基础的术后化疗可改善完全切除的Ⅲa期NSCLC患者的OS^[14, 24-27]^，但也有临床试验证实其心脏并发症及铂类相关的化疗毒性也可导致患者死亡。肺癌顺铂辅助化疗评估（LACE）的荟萃分析^[28]^明确了以顺铂为基础的辅助化疗可显著改善患者生存状况，辅助化疗组较未辅助化疗组5年生存率高出5.4%（HR 0.89, 95%CI: 0.82-0.96），且Ⅱ期-Ⅲa期患者为主要优势人群，但同时也发现，纳入研究的4, 584例患者中有19例（0.9%）因化疗药物相关而死亡。而氟尿嘧啶类药物（UFT）因其时间依赖的抗肿瘤作用，提高了术后患者的OS，且不良反应更少，被认为可用于术后长期辅助化疗。新型口服抗癌药S-1（替吉奥）为改良版UFT，降解更加缓慢，消化道毒性显著降低。一项来自日本的Ⅱ期临床试验对可切除的Ⅱ期-Ⅲa期NSCLC术后与S-1单药与顺铂联合S-1辅助化疗的疗效及安全性比较，证实了可切除的Ⅱ期-Ⅲa期NSCLC术后长期口服S-1单药较联合顺铂更可行，安全性更高^[29]^。近期另一个Ⅱ期研究同样证实了术后口服S-1单药相比于UFT能提高NSCLC患者的中位生存时间（92.4个月，95%CI: 45.5-139.3）并且毒性较小^[3]^。

Tecemotide是一种靶向粘蛋白1（MUC1）抗原疫苗，在不可切除Ⅲa/N2期NSCLC的治疗中的作用引发越来越多的重视。大型Ⅲ期随机化双盲临床研究START对比接受同步放化疗后的使用免疫治疗和安慰剂的生存，表明免疫治疗可延长同步放化疗后的OS^[30]^。但另一项大型Ⅲ期研究对比接受初始同步放化疗后的不可切除Ⅲa/N2期NSCLC患者使用tacemotide治疗和安慰剂治疗，结果并不支持START研究中OS延长的结论^[31]^。因此免疫治疗作为NSCLC的维持治疗是否有效仍在探索中。对于不可切除Ⅲa/N2期NSCLC，有新的Ⅲ期随机临床试验PACIFIC研究早期结果表明，相对于安慰剂，Durvalumab显著延长了同步放化疗后的PFS和OS^[32]^，也基于此研究结果，该免疫治疗在部分国家已获批用于放化疗后病情未进展的局部晚期不可切除NSCLC的治疗。即使在晚期NSCLC患者中，也有临床研究的早期结果表明化疗联合免疫治疗可能有更好的临床疗效。目前，Ⅲ期临床试验KEYNOTE 189和KEYNOTE 407研究正在对免疫治疗作为晚期NSCLC患者围手术期辅助治疗进行疗效研究。而对于可切除NSCLC，近期发表的一项研究在可切除的Ⅰ期、Ⅱ期或Ⅲa期NSCLC患者术前应用3 mg/kg的nivolumab，每2周静脉注射1次，在首剂后约4周进行手术。结果表明，免疫治疗有良好的安全性和耐受性，且以术后标本中残留肿瘤细胞 < 10%作为病理主要缓解标准，在20例完全切除的肿瘤中有45%患者达到此标准^[33]^。另一项正在进行的Ⅱ期单臂研究NADIM研究初步纳入30例患者，采用术前nivolumab和化疗3周期新辅助治疗，术后再予1年nivolumab单药辅助治疗。结果提示，病理完全缓解率达60%，主要缓解率达80%。以上数据和研究都表明，免疫治疗或可作为可切除NSCLC的新辅助治疗提高疗效、延长生存，但不同分期的亚组分析还待进一步研究。

## 5 结论

综合多项大型Ⅲ期临床研究结果提示（表 1），根治性手术切除在Ⅲa/N2期NSCLC患者的多学科治疗中为重要一环，其术式的选择需综合考虑肿瘤大小、淋巴结转移情况、术后不良反应等。三种治疗方式联合的方案可能优于两种治疗方式联合，但以铂类为基础的化疗联合手术或放疗这两种治疗方式对患者生存并无明显差别。其他化疗药物和靶向治疗、免疫治疗等也可能对部分Ⅲa/N2期NSCLC有效，有望替代化疗或联合化疗成为安全性更高的术后辅助治疗方案。最优方案还应根据患者个体情况评估治疗，且需要更多的前瞻性随机对照研究对综合治疗方案的优劣进行比较。

**1:** 关于Ⅲa/N2期NSCLC治疗方式的临床研究 List of clinical researches on treating Ⅲa/N2 NSCLC

Year	Research	Phase	*n*	Conclusion
Multidisciplinary treatment				
2006	ANITA	Ⅲ	840	Chemotherapy after surgery prolonged OS compared to placebo, especially in stage Ⅲ NSCLC
2008	ANITA	Ⅲ	840	Radiotherapy prolonged 5-year OS in stage pN2 NSCLC; Chemo-radiotherapy achieved longer OS than chemotherapy
2009	INT0139	Ⅲ	396	Chemo-radiotherapy significantly prolonged PFS and OS after surgery
2014	Aggarwal *et al*	Retrospective	219	Lobectomy after chemo-radiotherapy achieved long OS
2015	Pless *et al*	Ⅲ	232	Chemotherapy plus radical surgery has no significant difference in EFS and OS compared to trimodality treatment
Target therapy				
2014	SELECT	Ⅱ	100	Erlotinib prolonged DFS as 2 years after surgery and chemo-radiotherapy in Ia-Ⅲa NSCLC
2015	RADIANT	Ⅲ	161	Erlotinb prolonged DFS in mEGFR mutation after radical resection compared to placebo (*P* > 0.05)
2018	ADJUVANT	Ⅲ	222	Gefitinib plus chemotherapy prolonged mPFS in *EGFR* mutation after radical resection (*P* > 0.05)
2018	EVAN	Ⅱ	102	Erlotinib prolonged 2-/3-year DFS in stage ⅢA *EGFR* mutation after radical resection (*P* > 0.05)
Other therapies				
2008	Tsuboi *et al*	Ⅱ	24	S-1 prolonged OS in NSCLC after radical resection
2017	Okumura *et al*	Ⅱ	87	S-1 achieved better effects than S-1 plus chemotherapy in stage Ⅱ-Ⅲ NSCLC after surgery
2018	Forde *et al*	Ⅲ	20	Nivolumab achieved promising pathological response as neoadjuvant therapy
NSCLC: non-small cell lung cancer; EGFR: epidermal growth factor receptor; OS: overall survival; PFS: progression-free survival; DFS: disease-free survival; EFS: event-free survival.				

## References

[1] Bradbury P, Sivajohanathan D, Chan A (2017). Postoperative adjuvant systemic therapy in completely resected non-small-cell lung cancer: a systematic review. Clin Lung Cancer.

[2] Goldstraw P, Crowley J, Chansky K (2007). The IASLC Lung Cancer Staging Project: proposals for the revision of the TNM stage groupings in the forthcoming (seventh) edition of the TNM Classification of malignant tumours. J Thoracic Oncol.

[3] Tsuboi M, Kondo K, Takizawa H (2018). A feasibility study of postoperative adjuvant chemotherapy with fluoropyrimidine S-1 in patients with stage Ⅱ-ⅢA non-small cell lung cancer. J Med Invest.

[4] Decaluwe H, De Leyn P, Vansteenkiste J (2009). Surgical multimodality treatment for baseline resectable stage ⅢA-N2 non-small cell lung cancer. Degree of mediastinal lymph node involvement and impact on survival. Eur J Cardiothoracic Surg.

[5] Eberhardt WE, De Ruysscher D, Weder W (2015). 2^nd^ ESMO Consensus Conference in Lung Cancer: locally advanced stage Ⅲ non-small-cell lung cancer. Ann Oncol.

[6] Massard G, Renaud S, Reeb J (2016). N2-ⅢA non-small cell lung cancer: a plea for surgery!. J Thorac Dis.

[7] Betticher DC, Hsu Schmitz SF, Totsch M (2003). Mediastinal lymph node clearance after docetaxel-cisplatin neoadjuvant chemotherapy is prognostic of survival in patients with stage ⅢA pN2 non-small-cell lung cancer: a multicenter phase Ⅱ trial. J Clin Oncol.

[8] Mansour Z, Kochetkova EA, Santelmo N (2008). Persistent N2 disease after induction therapy does not jeopardize early and medium term outcomes of pneumonectomy. Ann Thoracic Surg.

[9] Douillard JY, Rosell R, De Lena M (2008). Impact of postoperative radiation therapy on survival in patients with complete resection and stage Ⅰ, Ⅱ, or ⅢA non-small-cell lung cancer treated with adjuvant chemotherapy: the adjuvant Navelbine International Trialist Association (ANITA) Randomized Trial. Int J Radiat Oncol Biol Physics.

[10] Pless M, Stupp R, Ris H-B (2015). Induction chemoradiation in stage ⅢA/N2 non-small-cell lung cancer: a phase 3 randomised trial. Lancet.

[11] Mauguen A, Pignon JP, Burdett S (2013). Surrogate endpoints for overall survival in chemotherapy and radiotherapy trials in operable and locally advanced lung cancer: a re-analysis of *meta*-analyses of individual patients' data. Lancet Oncol.

[12] Aggarwal C, Li L, Borghaei H (2014). Multidisciplinary therapy of stage ⅢA non-small-cell lung cancer: long-term outcome of chemoradiation with or without surgery. Cancer Control.

[13] Albain KS, Swann RS, Rusch VW (2009). Radiotherapy plus chemotherapy with or without surgical resection for stage Ⅲ non-small-cell lung cancer: a phase Ⅲ randomised controlled trial. Lancet.

[14] Douillard JY, Rosell R, De Lena M (2006). Adjuvant vinorelbine plus cisplatin versus observation in patients with completely resected stage IB-ⅢA non-small-cell lung cancer (Adjuvant Navelbine International Trialist Association [ANITA]): a randomised controlled trial. Lancet Oncol.

[15] van Meerbeeck JP, Kramer GW, Van Schil PE (2007). Randomized controlled trial of resection versus radiotherapy after induction chemotherapy in stage ⅢA-N2 non-small-cell lung cancer. J Natl Cancer Inst.

[16] McElnay PJ, Choong A, Jordan E (2015). Outcome of surgery versus radiotherapy after induction treatment in patients with N2 disease: systematic review and *meta*-analysis of randomised trials. Thorax.

[17] Eberhardt WE, Pottgen C, Gauler TC (2015). Phase Ⅲ study of surgery versus definitive concurrent chemoradiotherapy boost in patients with resectable stage Ⅲa (N2) and selected Ⅲb non-small-cell lung cancer after induction chemotherapy and concurrent chemoradiotherapy (ESPATUE). J Clin Oncol.

[18] Pennell NA, Neal JW, Chaft JE (2014). SELECT: A multicenter phase Ⅱ trial of adjuvant erlotinib in resected early-stage *EGFR* mutation-positive NSCLC. J Clin Oncol.

[19] Kelly K, Altorki NK, Eberhardt WE (2015). Adjuvant erlotinib versus placebo in patients with stage Ib-Ⅲa non-small-cell lung cancer (RADIANT): A randomized, double-blind, phase Ⅲ trial. J Clin Oncol.

[20] Zhong WZ, Wang Q, Mao WM (2018). Gefitinib versus vinorelbine plus cisplatin as adjuvant treatment for stage Ⅱ-ⅢA (N1-N2) *EGFR*-mutant NSCLC (ADJUVANT/CTONG1104): a randomised, open-label, phase 3 study. Lancet Oncol.

[21] Yue D, Xu S, Wang Q (2018). Erlotinib versus vinorelbine plus cisplatin as adjuvant therapy in Chinese patients with stage ⅢA *EGFR* mutation-positive non-small-cell lung cancer (EVAN): a randomised, open-label, phase 2 trial. The Lancet Respir Med.

[22] Dumont D, Do P, Lerouge D (2017). Off-label use of crizotinib as a neoadjuvant treatment for a young patient when conventional chemotherapy gave no benefits in stage Ⅲa non-small cell lung cancer. Am J Case Reports.

[23] Imanishi N, Yoneda K, Taira A (2018). Major pathologic response to alectinib in ALK-rearranged adenocarcinoma of the lung. Surg Case Rep.

[24] Arriagada R, Bergman B, Dunant A (2004). Cisplatin-based adjuvant chemotherapy in patients with completely resected non-small-cell lung cancer. N Engl J Med.

[25] Winton T, Livingston R, Johnson D (2005). Vinorelbine plus cisplatin *vs* observation in resected non-small-cell lung cancer. N Engl J Med.

[26] Ou W, Sun HB, Ye X (2010). Adjuvant carboplatin-based chemotherapy in resected stage ⅢA-N2 non-small cell lung cancer. J Thoracic Oncol.

[27] Van Zandwijk N, Smit EF, Kramer GW (2000). Gemcitabine and cisplatin as induction regimen for patients with biopsy-proven stage ⅢA N2 non-small-cell lung cancer: a phase Ⅱ study of the European Organization for Research and Treatment of Cancer Lung Cancer Cooperative Group (EORTC 08955). J Clin Oncol.

[28] Pignon JP, Tribodet H, Scagliotti GV (2008). Lung adjuvant cisplatin evaluation: a pooled analysis by the LACE Collaborative Group. J Clin Oncol.

[29] Okumura N, Sonobe M, Okabe K (2017). Feasibility of adjuvant chemotherapy with S-1 plus carboplatin followed by single-agent maintenance therapy with S-1 for completely resected non-small-cell lung cancer: results of the Setouchi Lung Cancer Group Study 1001. Int J Clin Oncol.

[30] DeGregorio M, Soe L, Wolf M (2014). Tecemotide (L-BLP25) versus placebo after chemoradiotherapy for stage Ⅲ non-small cell lung cancer (START): a randomized, double-blind, phase Ⅲ trial. J Thorac Dis.

[31] Katakami N, Hida T, Nokihara H (2017). Phase Ⅰ/Ⅱ study of tecemotide as immunotherapy in Japanese patients with unresectable stage Ⅲ non-small cell lung cancer. Lung Cancer.

[32] Antonia SJ, Villegas A, Daniel D (2018). Overall survival with durvalumab after chemoradiotherapy in stage Ⅲ NSCLC. N Engl J Med.

[33] Forde PM, Chaft JE, Smith KN (2018). Neoadjuvant PD-1 blockade in resectable lung cancer. N Engl J Med.

